# Supplementary material for: Functional Connectivity between Task-Positive Networks and the Left Precuneus as a Biomarker of Response to Lamotrigine in Bipolar Depression: A Pilot Study
Source: Pharmaceuticals (Basel). 2021 Jun 3;14(6):534. doi: 10.3390/ph14060534 (PMC8229811; doi:10.3390/ph14060534)
Supplement: Supplementary file 1 [file pharmaceuticals-14-00534-s001.zip › pharmaceuticals-1197586-supplementary.pdf]

Table S1. Medications and their daily doses taken by patients with bipolar depression; responder/non-responder status at 10-12 weeks is shown. NB the dose had been unchanged for at least 6 months before patients entered the study.

Patients	Medications and their daily doses at the baseline scan
**Responders**	
1	Aripiprazole 10mg
2	Quetiapine 150mg
3	Quetiapine 100mg<br>Fluoxetine 20mg
4	Quetiapine 800mg<br>Lithium 1200mg
5	Duloxetine 20mg<br>Lithium 600mg
6	Venlafaxine 75mg
7	Venlafaxine 75mg
8	Fluoxetine 20mg
9	Citalopram 20mg
10	None
11	None
12	None
13	None
14	None
15	None
**Non-responders**	
16	Sertraline 200mg
17	Quetiapine 500mg<br>Venlafaxine MR 75mg
18	Lithium 1200mg
19	Mirtazapine 30mg<br>Aripiprazole 20mg<br>Sodium valproate 1600mg
20	None
21	None

Table S2. Correlations between established functional connectivity measures (parameter estimates) and clinical scores (% change baseline vs. post-treatment).

Correlations			HAMD	BDI	STAI
			% change	% change	% change
**Pearson's**	**DAN with**	Correlation Coefficient	-0.704	-0.529	-0.151
**r**	**left precuneus**	Sig. (2-tailed)	0.001	0.02	0.525
		N	20	19	20
	**Right FPN with**	Correlation Coefficient	-0.639	-0.523	-0.349
	**left precuneus**	Sig. (2-tailed)	0.002	0.022	0.132
		N	20	19	20
	**Left FPN with**	Correlation Coefficient	-0.391	-0.413	-0.205
	**left ITG/fusiform/latOCC**	Sig. (2-tailed)	0.089	0.079	0.386
		N	20	19	20
	**SSMN with**	Correlation Coefficient	-0.781	-0.538	-0.247
	**left hippocampus**	Sig. (2-tailed)	<0.001	0.018	0.294
	**/ amygdala**	N	20	19	20
		Correlation Coefficient	-0.616	-0.425	-0.37
	**DMN with**	Sig. (2-tailed)	0.004	0.07	0.109
	**Left precuneus**	N	20	19	20
